# Detection of Early Onset Carnitine Palmitoyltransferase II Deficiency by Newborn Screening: Should CPT II Deficiency Be a Primary Disease Target?

**DOI:** 10.3390/ijns7030055

**Published:** 2021-08-13

**Authors:** Rachel Mador-House, Zaiping Liu, Sarah Dyack

**Affiliations:** 1 IWK Health, Halifax, NS B3K 6R8, Canada; rachelmador@hotmail.com (R.M.-H.); zaiping.liu@iwk.nshealth.ca (Z.L.); 2 Department of Pathology and Laboratory Medicine, Dalhousie University, Halifax, NS B3H 4R2, Canada; 3 Departments of Paediatrics and Medicine, Dalhousie University, Halifax, NS B3H 4R2, Canada

**Keywords:** carnitine, CPT II deficiency, transferase, newborn, screening, treatment

## Abstract

Early-onset carnitine palmitoyltransferase II deficiency (CPT II deficiency) (OMIM 600650) can result in severe outcomes, which are often fatal in the neonatal to infantile period. CPT II deficiency is a primary target in the Maritime Newborn Screening Program. We report a case of neonatal-onset CPT II deficiency identified through expanded newborn screening with tandem mass spectrometry. Identification through newborn screening led to early treatment interventions, avoidance of metabolic decompensation, and a better clinical outcome. Newborn screening for CPT II deficiency is highly sensitive and specific with no false positives identified. The only screen positive case detected identified a true positive case. This experience illustrates the importance of newborn screening for CPT II deficiency and demonstrates why reconsideration should be taken to add this disease as a primary newborn screening target.

## 1. Introduction

Carnitine palmitoyltransferase II deficiency (CPT II deficiency) (OMIM 600650) is an autosomal recessive disorder of fatty acid metabolism. Three clinical phenotypes have been described, characterized by differing ages of onset and organ involvement: early onset forms include both severe neonatal lethal and severe infantile forms, and a later onset mild myopathic adult form [1,2]. Patients with the early-onset forms commonly experience severe outcomes, which are often lethal [3,4]. CPT II deficiency can be detected using tandem mass spectrometry leading to early identification and diagnosis [5,6]. In this report, we describe a favorable outcome in a patient with a form of CPT II deficiency who developed symptoms in infancy, detected through expanded newborn screening.

**Figure 1:**
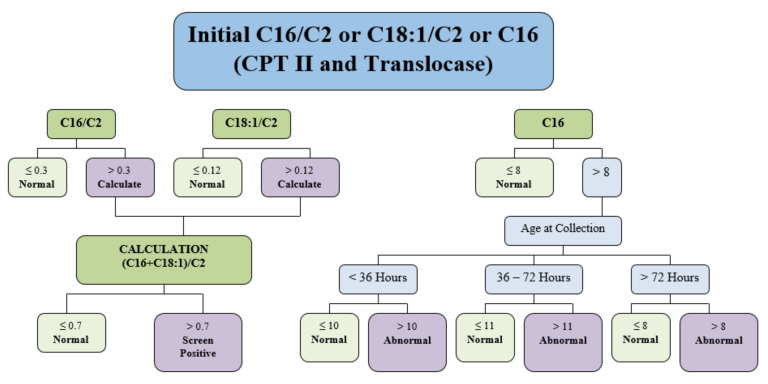
Algorithm used in 2014 for Newborn Screening for CPT II deficiency and CACT deficiency. The logic in this figure has since been updated. Currently, (C16 + C18:1)/C2 is the primary screening biomarker with a cut-off of >0.7 as screening positive. All acylcarnitine values are in μmol/L.

The MS/MS-based metabolic NBS was introduced in 2000 via the API 2000 platform from Sciex. The Maritime Newborn Screening Program (MNBSP) began screening for CPT II deficiency in April of 2005 using the screening algorithm illustrated in Figure 1 [7]. Since then, over 220,000 newborns have been screened and only one screen positive case has been identified, which we report here. There have been no false positive cases identified. CPT II deficiency is classified as a Secondary Condition in the Recommended Uniform Screening Panel (RUSP) in the United States of America [8] however, based on the high sensitivity and specificity in our experience, we suggest that it should be considered for addition as a primary target on newborn screening panels.

## 2. Case Report

The patient was a 3374 g Caucasian female infant who was born at 40 weeks and 3 days via spontaneous vaginal delivery. The pregnancy was followed by a Maternal Fetal Medicine Team due to an area of placental abnormality identified on early ultrasound. No other ultrasound anomalies were identified. The family history was noncontributory, and parents were both Caucasian, healthy and nonconsanguineous. In the neonatal period, she had mild jaundice, but did not require phototherapy and was discharged home after 2 days. No dysmorphic features nor congenital anatomical anomalies were noted.

On day 8 of life, she was identified as at-risk for CPT II deficiency or carnitine-acylcarnitine translocase (CACT) deficiency via expanded newborn screening using tandem mass spectrometry. The full screening algorithm for CPT II deficiency and/or CACT deficiency can be found in Figure 1.

Analysis of her dried blood spot, collected at 25 h after birth, revealed abnormalities as outlined in Table 1.

Plasma (collected at 9 days of age) carnitine/acylcarnitine profile analysis was performed in the Department of Pediatric Laboratory Medicine, The Hospital for Sick Children, Toronto, Canada, and it confirmed elevations in long chain acylcarnitines consistent with CPTII deficiency.

Analytes listed in Table 1 were used to evaluate the risk of this infant for CPT II deficiency. Analytes measures for the evaluation of other diseases present on the newborn screening panel are not included. Only the (C16 + C18:1)/C2 and C0/(C16 + 18) ratios were used in the screening algorithm for CPT II then. Other analytes were included as a profile interpretation to facilitate clinical care.

She was clinically assessed and had a normal physical examination on day 9 of life. Specifically, she had a normal cardiac assessment, normal tone, and no evidence of hepatosplenomegaly. She was breast fed every 2 to 3 h. She had a normal glucose, ammonia, and venous blood gas pH. Her lactate was elevated at 6.7 mmol/L. Repeat lactate levels remained mildly elevated (2.3–3.3 mmol/L). Lactate testing after age 3 years revealed normal values. Her creatine phosphokinase level (CPK) was mildly elevated at 249 μ/L; reference range 0–130 μ/L. The parents were given recommendations for the care of their infant; to avoid fasting for periods longer than 3–4 h, to bring her to a local emergency hospital if she presented with illness, lethargy, seizures or if she was unable to feed every 3 h, and they were given an emergency protocol letter.

A diagnosis of CPT II deficiency was confirmed after residual carnitine palmitoyltransferase II enzyme activity was reported as 5% of normal [9] and genetic testing for CACT was negative. The laboratory director who issued the CPT II report suggested that her low residual enzyme activity placed her within the severe infantile hepato-cardio-muscular spectrum of the CPT II disorder. She was found to have two DNA variants in *trans* in the *CPT2* gene, c.149C>A, p.Pro50His (dsSNP reference: rs28936375) and c.1369A>T, p.Lys457Ter (dbSNP reference: none).

She has had approximately 15 hospital admissions due to episodes of decreased feeding and/or vomiting during her first 5 years of life. Her highest CPK during a hospital admission was 49,945 μ/L at age 1 year after <24 h of low-grade fever secondary to a viral upper respiratory tract infection. Although she has had hospital admissions where she did not develop an elevated CPK, typically her CPK has been in the 1000 s μ/L range. She had one episode at age 4 years, when she was admitted with fever secondary to otitis media where her CPK reached 6826 μ/L and despite being treated with amoxicillin with dissolution of fever within 48 h, her CPK took days to resolve, and we speculated that this was due to her having post-tussive emesis and inadequate caloric intake. Typically, she received IV infusions containing D10 and normal saline during admission for intercurrent illness. Her parents stated that when she was ill with vomiting and muscle pain, both ceased quickly once she had an IV containing D10. Because of her frequent admissions, her local care team developed a protocol to directly admit her to the pediatric floor of the hospital and administer D10 IV fluids, in order to expedite timely treatment. In this protocol, her CPK needed to be less than 300 μ/L before her IV D10 was weaned and prior to discharge home, because if her IV was removed before that goal, her muscle pain and elevated CPK would recur.

Management of the patient included a low-fat diet, carnitine supplementation, walnut oil, vitamin D supplementation, avoidance of fasting, cornstarch with milk at bedtime, avoidance of metabolic stressors such as infection, avoidance of ibuprofen due to a known association of increased CPK levels with ibuprofen use in CPT II deficiency, and an emergency management protocol should she fall ill. Medium chain triglyceride (MCT) oil was prescribed at age 6 months, and she took MCT until she was 3 years of age. The patient could not tolerate MCT at that time because of the taste. Parents tried MCT oil, Liquigen, and MCT-based infant formula as alternate ways to provide MCT, but patient would not accept them or experienced episodes of emesis after ingestion. Walnut oil was an important source of essential fatty acids as the patient followed a low-fat diet. She was non-fasting overnight until 2 years of age. When strict control of fasting overnight was lessened, she would wake up famished in the night, so cornstarch was added to her bedtime snack. At that time, overnight fasting bloodwork was performed, which was normal and confirmed that she could safely fast 12 h and maintain normal glucose and CPK levels. She has had annual cardiovascular assessments, including echocardiography and ECG, which have been normal. A 24 h Holter monitor assessment performed at age 4 was normal. An indwelling port was inserted at age 4 years to improve venous assess. Recurrent muscle pain and fatigue when well, which resolved with rest and fluids, was problematic. These symptoms prevented her from participating in typical childhood activities including trick or treating for Halloween at age 4 years where she developed pain and fatigue when walking a relatively short distance and needed to be carried.

On a clinical review at age 4 years, a revisiting of her diet and MCT supplementation was undertaken. Her diet was shifted to contain a greater proportion of lean protein. She now receives 1 sachet of MCT procal (10 g of MCT) in the morning, and another is taken prior to participation in physical activity, with a subsequent improvement in her exercise capacity. She spent a successful week at the Disney parks, she attends a full-time kindergarten class and can participate in dance classes, acrobatics, soccer, and swimming, which she would not have been able to do in the past. She eats frequently, about every 2 h, and no longer complains of muscle pain. Better treatment of her asthma is preventing coughing with vomiting during illnesses. When she is unwell, she is eating more, especially protein, and the family finds that this made a substantial difference in her response to illness. She has normal intelligence and has not been admitted for an intercurrent illness in the past year.

## 3. Discussion

There are three reported CPT II deficiency phenotypes: a mild adult-onset myopathic form, a severe infantile form, and a lethal neonatal form. Without proper diagnosis and treatment, all infants with neonatal lethal form will die, most before the age of 6 months, and many with severe infantile form will die or have serious permanent organ and brain damage [1,2]. The importance of early diagnosis and treatment of fatty acid oxidation defects by newborn screening has been established [10]. Past reports suggest that early treatment can prolong survival in patients with neonatal and infantile forms of CPT II deficiency [5,11]. Our patient’s presentation does not fit neatly into this classification system as will be more fully described below. It is possible that early detection and treatment may have altered her disease progression, with her case illustrating the outcome after early diagnosis and treatment.

MNBSP uses tandem mass spectrometry on dried blood spot specimens collected between 24–48 h of life to screen for CPT II deficiency. Our patient’s sample was collected at 25 h of life. A newborn screening blood sample to screen for CPT II deficiency is ideally collected on day 2 to 3 of life, as due to maximal catabolism, it is the most sensitive time to detect an increase in acylcarnitines [12]. Our algorithm has been modified since the time of our patient’s birth. Specimens are determined to be screen positive for CPT II deficiency if (C16 + C18:1) /C2 > 0.7. The patient’s newborn screening result was (C16 + C18:1)/C2 = 1.46, which was double our cut off for screen positive samples. The (C16 + C18:1)/C2 ratio cannot differentiate between CPT II and carnitine-acylcarnitine translocase (CACT) deficiencies as both of these conditions may elevate the (C16 + C18:1)/C2 above 0.7 [13]. This means that newborn screening for CPT II deficiency will indicate that an infant is at-risk for both of these conditions. Residual enzyme activity assays and genetic analysis were used to differentiate between these diseases and confirm a diagnosis [14]. Our patient showed free, total, and acyl-carnitines levels at the low end of the reference range, consistent with the patient’s mother’s low carnitine levels, which were caused by her diet.

The diagnosis of CPT II deficiency was confirmed in this patient through residual CPT II enzyme assay and gene variant analysis. Residual CPT II enzyme activity was evaluated in fibroblasts and was found to be 5% of normal. CPT II enzyme activity levels less than 10% are typically associated with the severe infantile hepato-cardio-muscular form of CPT II, which is characterized as presenting within the first year of life [15]. Our patient, however, has not manifested hepatomegaly and has had normal cardiac evaluations despite having a low enzyme activity. It is unclear if our patient does not manifest the severe infantile hepato-cardio-muscular form of CPT II because she has a milder form of the condition or if early intervention changed the course of her disease.

The molecular analysis of *CPT2* identified two variants in *trans*; c.149C>A, p.Pro50His (dsSNP reference: rs28936375) and c.1369A>T, p.Lys457Ter (dbSNP reference: none). The first variant is in exon 1 and has been reported to be associated with CPT II deficiency when homozygous or when heterozygous in *trans* with a second deleterious variant [4,16,17]. This variant is postulated to disrupt the shuttling of the palmitoylcarnitine substrate into the active site of the CPT II enzyme [18]. Interestingly this variant was found to be associated with 25% residual activity when homozygous in fibroblasts [16] and has been associated with the “adult” form of the disease when in *trans* with another variant [4]. The second variant is in exon 4 and changes the lysine at amino acid position 457 to a STOP codon. This is predicted to cause a premature truncation of the CPT II polypeptide. This variant has been reported in association with severe infantile hepato-cardio-muscular form in the literature [19]. The patient’s parents were found to be heterozygous carriers confirming that our patient inherited these variants in *trans*. These molecular results confirm that the decrease in CPT II enzyme activity was caused by a reduction of CPT II enzyme production and/or function.

Based on the residual CPT II enzyme activity of 5%, compound heterozygous pathogenic variants, and her clinical presentation with early onset muscle pain without cardiac or liver involvement, this patient did not clinically present as one of the classic forms of CPT II deficiency. Currently she would be clinically described as having myopathic disease presenting within the first year of life. The mild adult myopathic form of CPT II can present in childhood with myopathic symptoms reported as occurring even as young as 1 year of age [19]; therefore, the phenotypic description of the mild myopathic form being the adult form is misleading. Indeed, it could be argued that CPT II deficiency presents as a continuum of severity rather than three discrete phenotypes. In a patient described by Vlaitudu [4] who presented with symptomatic hypoglycemia in the first year of life, the Pro50His “adult” variant and a frameshift variant, Q413fs, were discovered. This patient was found to have 17% activity of the CPT II enzyme in cultured fibroblasts and was described as having an atypical late onset infantile phenotype. Our patient, who also has the Pro50His variant in concert with a severe truncating variant, presented with a similar atypical phenotype of early onset symptomatic myopathy responding to IV therapy. Perhaps we prevented severe hypoglycemia with early detection via newborn screening and prevention of fasting during illness. Although we will not know what the outcome would have been for our patient without newborn screening, it remains possible that her disease could have been fatal, in contrast to the expected outcome for the mild myopathic phenotype.

Treatment of CPT II deficiency consists of avoidance of known triggers (certain medications, illness, extreme cold, and extreme heat), administration of intravenous glucose and carnitines, prevention of catabolism during illness, avoidance of fasting, a high-carbohydrate and low-fat diet, and supplementation of medium chain triglycerides and vitamin D [10,20]. There have been reports of medication-induced side effects in individuals with CPT II deficiency. Agents that should be avoided include valproic acid, general anesthesia, ibuprofen, and diazepam in high doses [2,21,22]. This patient had episodes of elevated CPK levels during illness and muscle pain occasionally during activity. IV fluids and rest have resolved all illness-related events. Ceasing activity, resting, and ingestion of fruit juice resolved all episodes of muscle pain. Waking at night with hunger was relieved with cornstarch ingestion at bedtime. The simultaneous increase of MCT in her diet along with an increase in her dietary protein, coincided with a reduction of illness associated admissions to hospital and muscle pain and fatigue. It is possible that consistent administration of MCT oil contributed to the improvement in her symptoms. Although we cannot prove that the addition of increasing amounts of dietary protein improved her symptoms, it may be worth more study.

Expanded newborn screening for CPT II deficiency accurately identified the only known infant at-risk for CPT II deficiency in our Canadian Maritime Provinces, allowing for presymptomatic treatment. For this patient, early identification allowed for early supplementation and risk avoidance recommendations. It also allowed for early education regarding actions to be taken during illness. The patient’s local hospital, with the help of the Metabolic service, was able to create an emergency protocol and establish a timely treatment plan before the patient’s first hospitalization, leading to decreased stress and decreased time-to-treat for her first hospitalization. Early identification also supported early cardiac evaluations and close monitoring. Due to these early interventions, dietary support has been managed efficiently and changes to her clinical status have been monitored closely.

The RUSP lists 35 conditions listed as primary state newborn screening targets. There are 26 secondary targets that could be detected as they are differential diagnoses of a primary target. In Canada, there are no official uniform screening panel recommendations, therefore provincial newborn screening programs evaluate and prioritize conditions to be added as primary or secondary targets independently. MNBSP’s experience screening for CPT II deficiency since 2005 includes only one sample identified as screen positive, with a subsequent confirmation of CPT II deficiency. There have been no false positive cases. The Medical Genetics team, which is the only referral center in the Maritime Provinces for inborn errors of metabolism, has not diagnosed nor been made aware of any cases of CPT II deficiency that were missed by the newborn screen since 2005, although there could be mild myopathic CPT II deficiency patients who have yet to present clinically. The screening analysis, performed using tandem mass spectrometry, is used to screen for other inborn errors of metabolism, and therefore the addition of CPT II deficiency introduced a minimal cost in both the laboratory and in the clinical assessment and follow up of screen positive cases. And finally, early diagnosis in this patient led to what we consider to be significant and successful clinical interventions. MNBSP suggests the early onset CPT II deficiency to be considered as a primary target for newborn screening programs. In our small program, we found screening for CPT II deficiency to have high accuracy, sensitivity, and specificity. The available evidence, though limited, supports early diagnosis and treatment having a beneficial clinical impact. 

## 4. Conclusions

Expanded newborn screening correctly identified a patient with infantile onset of CPT II deficiency. As a consequence of the newborn screening, we suggest early treatment and timely intervention during illness likely prevented hypoglycemia and may have contributed to her normal neurological development and absence of cardiac involvement. Larger centers with more screening data may find our results interesting and may consider evaluating early onset CPT II deficiency as a primary target for newborn screening programs. We found that screening had acceptable accuracy, sensitivity, and specificity on a technological platform used to evaluate other inborn errors of metabolism, and that early diagnosis leading to expedited treatment may have allowed for a better clinical outcome.

## Figures and Tables

**Table 1:** Relevant free carnitine (C0) and acylcarnitine levels from newborn screening (NBS) dried blood spot (DBS) and initial plasma acylcarnitine profile.

Analyte	Level-DBS (μmol/L)	Reference Range (DBS)	NBS Cut Off	Plasma Level (μmol/L)	Reference Range (Plasma)
**(C16 + C18:1)/C2**	1.46	0.13–0.17	>0.70	0.54	NA
**C0/(C16 + C18)**	1.23	7.33–11.59	<2.98	8.71	NA
**C16**	5.44	0.46–6.20	>10.00	1.01	<0.31
**C16/C2**	1.08	0.08–0.13	>0.30	0.24	NA
**C18:1/C2**	0.38	0.06–0.04	>0.12	0.30	NA
**C3/C16**	0.03	0.87–0.81	NA	0.35	NA
**C14/C3**	2.94	NA	NA	1.4	NA
**C0**	8.26	8.00–58.00	NA	12.2	12.0–60.0
**C2**	5.02	6.00–49.00	NA	4.22	4.65–35.39
**C3**	0.18	0.40–5.00	NA	0.35	<1.08
**C14**	0.53	0.07–0.56	NA	0.49	<0.11
**C16OH**	0.05	0.01–0.10	NA	0.05	<0.05
**C18:1**	1.89	0.33–2.20	NA	1.28	<0.28
**C18**	1.28	0.23–1.71	NA	0.39	<0.10

## Data Availability

The data presented in this study are available in entirety within this article.
